# *AHR* rs4410790 genotype and IgG levels: Effect modification by lifestyle factors

**DOI:** 10.1371/journal.pone.0290700

**Published:** 2023-10-02

**Authors:** Jaewon Khil, Soyoun Kim, Minhyeong Lee, Hyeonmin Gil, Seok-Seong Kang, Dong Hoon Lee, Youngeun Kwon, NaNa Keum

**Affiliations:** 1 Department of Food Science and Biotechnology, Dongguk University, Seoul, Korea; 2 Department of Nutrition, Harvard T.H. Chan School of Public Health, Boston, Massachusetts, United States of America; 3 Department of Biomedical Engineering, Dongguk University, Seoul, Korea; 4 Department of Sport Industry Studies, Yonsei University, Seoul, Republic of Korea; Sungkyunkwan University and Samsung Advanced Institute of Health Science and Technology (SAIHST), REPUBLIC OF KOREA

## Abstract

Inflammation is a multifaceted marker resulting from complex interactions between genetic and lifestyle factors. Emerging evidence suggests Aryl hydrocarbon receptor (AHR) protein may be implicated in the regulation of immune system and inflammatory responses. To investigate whether rs4410790 genotype (TT, TC, CC) near *AHR* gene is related to serum IgG levels, a marker of chronic inflammation, and whether lifestyle factors modifies the relationship, we conducted a cross-sectional study by recruiting 168 Korean adults. Participants responded to a lifestyle questionnaire and provided oral epithelial cells and blood samples for biomarker assessment. Among these participants, C allele was the minor allele, with the minor allele frequency of 40%. The rs4410790 TT genotype was significantly associated with elevated IgG levels compared with TC/CC genotypes, after adjusting for potential confounders (p = 0.04). The relationship varied significantly by levels of alcohol consumption (P interaction = 0.046) and overweight/obese status (P interaction = 0.02), but not by smoking status (P interaction = 0.64) and coffee consumption (P interaction = 0.55). Specifically, higher IgG levels associated with the TT genotype were evident in frequent drinkers and individuals with BMI≥23kg/m^2^, but not in their counterparts. Thus, rs4410790 genotype may be associated with IgG levels and the genetic predisposition to higher IgG levels may be mitigated by healthy lifestyle factors like infrequent drinking and healthy weight.

## Introduction

The Aryl hydrocarbon receptor (*AHR*) gene on chromosome 7 encodes AHR protein [1], a ligand-activated transcription factor that modulates responses to xenobiotics (e.g., dioxins) [2]. In recent years, a new role of AHR has been increasingly recognized [3]. Expressed in diverse immune cells including macrophages, NK cells, B lymphocytes, AHR has been suggested to play a key role in regulating immune system and inflammatory responses [3, 4]. Thus, genetic variants of the *AHR* gene may have consequences on immune and inflammatory responses. One candidate is rs4410790, a single-nucleotide polymorphism (SNP) near the *AHR* gene (more specifically, located at 7p21, 54 kb upstream of AHR on chromosome 7). The rs4410790 variants were shown to be associated with cerebellum *AHR* gene methylation, thereby affecting the level of its genetic expression [1]. In genome-wide association studies of European descendants, rs4410790 C allele compared to T allele was associated with higher consumption of caffeine [5–7], which has a myriad physiological effect including immunomodulatory actions and anti-inflammatory effect [8]. Yet, a direct association between rs4410790 and inflammatory markers has been rarely explored.

Immunoglobin G (IgG), the most abundant (about 70–80%) antibody in the blood, plays a central role in the immune system [9]. After initial bacterial or viral infection or other antigen exposure, IgG is produced in a delayed timescale by plasma B cell [9]. The IgG activates Fcγ-receptors in the surface of immune cells, which produce and release proinflammatory cytokines [10, 11]. Thus, an elevated IgG level is a marker of chronic active infection or inflammation. Interestingly, emerging evidence suggests that AHR may affect the production of IgG by mediating the effect of AHR’s ligands on immunoglobin productions [12]. In various animal models, 2,3,7,8-tetracholorodibenzo-p-dioxin, a ligand of AHR, was shown to inhibit the differentiation of B-lymphocyte into antibody-forming cells and consequently to suppress immunoglobin expression including IgG [12]. A role of AHR in modulating IgG expression is also suggested in the observation that decreased AHR levels induced lower IgG expression [13]. Thus, rs4410790 near the *AHR* gene may be linked to circulating IgG levels.

Of note, circulating levels of IgG are influenced not only by antigen exposures, but also by lifestyle factors that are associated with immune and inflammatory responses such as alcohol drinking [14], smoking [15], and obesity [16]. In a study of 460 adults in Spain, serum IgG levels were lower in moderate drinkers than in light or heavy drinkers, in smokers than non-smokers, and in normal weight individuals than in obese people[17]. Considering that not only AHR but also drinking, smoking, and obesity are associated with circulating IgG levels, rs4410790 and the lifestyle factors might interact in modulating the IgG levels. Thus, in addition to investigating the relationship between rs4410790 genotype (TT, TC, CC) and IgG levels, we examined whether the aforementioned lifestyle factors such as alcohol consumption, overweight/obese and smoking modified the relationship. In addition, the variants of rs4410790 have been most widely investigated in relation to caffeine intake [5–7] and coffee is known for its anti-inflammatory effect [18]. Thus, we also explored the role of coffee consumption in mediating and modifying the relationship between rs4410790 genotype (TT, TC, CC) and IgG levels.

## Materials and methods

### Study participants

From March to May in 2019, a cross-sectional study was conducted in Dongguk University, Korea by recruiting healthy individuals aged 18 years or older. All participants graduated from high school and were of legal age to drink and smoke [19]. At first, 200 subjects applied for the study participation, but 20 individuals were excluded due to one of the following reasons: with a history of major diseases or surgery, on regular treatment or prescription medicine, with mental illness or anemia, unable to exercise, or pregnant or planning for it. 180 individuals (119 men, and 61 women) visited the research center for the collection of oral epithelial cells, blood draw, questionnaire response, and anthropometric measurements. We further excluded 12 individuals whose serum were contaminated by hemolysis. The analytic cohort for this study consisted of a total of 168 individuals (110 men, 58 women).

Before participation in our study, participants were given full explanation about the study and granted the right to opt out of the study at any time during the study. Participants provided written informed consents before enrollment to the study and received a gift voucher as a compensation for the study participation. The study protocol was reviewed and approved by the Institutional Review Board of the Dongguk University Ilsan Hospital (IRB number: 2018-12-006-010). We conducted this study in accordance with the Helsinki Declaration principles.

### Identification of rs4410790 genotype

ExgeneTM Tissue SV (GeneAll, Seoul, Korea) was used to extract DNA from buccal swabs. A SNP array (Theragen PMRA, Seoul, Korea) was performed to identify genotype of rs4410790. The procedures of genotype analysis and quality control are described in detail in a previous study [20]. Accuracy of genotype based on the SNP array was demonstrated to be 0.94 in an Asian population for SNPs with minor allele frequencies > 5%. The call rate was ≥97% and the distribution of TT, TC, CC was in Hardy-Weinberg equilibrium (*χ*^2^ test, P>0.05).

### Assessment of serum IgG levels

Serum IgG levels were measured by arrays of protein G and A at different concentrations [21, 22]. Immunoassays were performed using the microarrays as follows; each chamber was treated with 2% BSA in PBST for 1 h, washed with PBST, and incubated with 200 nL of diluted human serum (1/400) in PBST for 1 h. The microarray was then washed with PBST and treated sequentially with a solution of detection antibody and a solution of Cy3-conjugated goat anti-human IgG in PBST for 1 h. The fluorescence intensity was measured using a GenePix 4000B (Axon Instruments, Union city, CA) using an excitation wavelength of 532 nm and an emission wavelength of 570 nm. The IgG signal intensity, which was denoted in arbitrary unit (A.U.), was determined by calculating the average of triplicates.

### Assessment of covariates

Information on factors that could influence the level of serum IgG levels were collected in various methods. For demographic and lifestyle information, online frequency questionnaire was administered. The list includes age, sex, alcohol consumption (hardly ever, 1–2 times/week, 3–4 times/week, 5–6 times/week, every day), coffee consumption (hardly ever, 1–2 times/week, 3–4 times/week, 5–6 times/week, every day), current smoking status (yes, no), daily aerobic exercise (<30 minutes, ≥30 minutes–<1 hour, ≥1hour).

In light of the evidence that obesity in childhood and adolescence initiates metabolic inflammation that has a lasting influence on the risk of adult disease [23], we also collected information on body shape during high school.

To reflect total vitamin D reserves in the body, serum levels of 25-hydroxyvitamin D (25(OH)D) were measured using the blood drawn for IgG measurement.

To calculate current body mass index (BMI), weight in kilograms divided by the square of the height in meters (*kg*/*m*^2^), height and weight were measured using bioelectrical impedance analyzer (Inbody720, Seoul, Korea) in light clothing and bare feet. By the World Health Organization, overweight and obese for Asians are defined as 23kg/m^2^≤BMI<25 kg/m^2^ and BMI≥25kg/m^2^, respectively [24]. These cut-offs account for the fact that Asians have a higher fat mass for a given BMI compared to other racial groups [25].

### Statistical analysis

The χ2 test for categorical variables and t-test for continuous variables were used to compare baseline characteristics of the participants according to rs4410790 genotype.

Linear regression was performed to examine the relationship between rs4410790 genotype and serum IgG level, with multivariable regression adjusting for potential confounders described in the covariate section. To examine the degree to which the association is mediated through the effect of rs4410790 genotype on caffeine, we ran another multivariable linear regression by further adjusting for coffee intake, a major source of caffeine intake.

Subgroup analyses by levels of drinking, overweight/obese, smoking, and coffee consumption were performed to explore whether the relationship between rs4410790 genotype and serum IgG level differs by these stratifying variables. Potential interactions between rs4410790 genotype and the stratifying variables were tested by adding their cross-product terms to the multivariable linear regression and running the Wald test on the terms.

P-value of < 0.05 was considered statistically significant. All statistical analyses were conducted using SAS 9.4 (SAS Institute, Cary, NC, USA).

## Result

### Baseline characteristics of participants by rs4410790 genotype

Characteristics of the 168 participants are presented according to rs4410790 genotype (T>C) in Table 1. For each genotype of TT, TC, and CC, there were 58, 85, 25 subjects, respectively. In this population, C allele was the minor allele, with the minor allele frequency of 40%. There was no trend of IgG levels with increasing number of C allele.

**Table 1:** Characteristic of the participants according to rs4410790 genotype.

	Genotype		P value
	TT	TC/CC	
Number of subjects, %	35	65	
Age, years, mean (SD)	21 (2)	22 (3)	0.32
Men, (%)	50	43	0.37
Alcohol consumption, time/week (%)			
<1	90	86	0.44
≥1	10	14	
Coffee consumption, time/week (%)			
<1	41	33	0.30
≥1	59	67	
Overweight/obese (BMI≥23kg/m²), (%)			
No	55	66	0.15
Yes	45	34	
Overweight/obese in high school, (%)			
No	72	76	0.57
Yes	28	24	
Current smoking status, (%)			
No	83	85	0.76
Yes	17	15	
Aerobic exercise per day, (%)			
<30 min	12	25	0.13
30 min ‐ < 1 hour	50	47	
≥1 hour	38	28	
25(OH)D level in blood, mean (SD)	10 (5)	11 (6)	0.30

Abbreviation: SD, standard deviation; BMI, Body Mass Index; min, minute

Data are presented as mean (standard deviation) for continuous variables and proportion for categorical variables.

The mean age of the participants was 21 years (range: 18–24 years). Compared to participants with TT genotype, those with TC/CC genotype were more likely to be women, to drink alcohol, to drink coffee, and to have higher levels of circulating 25(OH)D. However, they were less likely to be overweight/obese currently and in high school, to smoke, and to exercise. Yet, the differences in baseline characteristics were not statistically significant (Table 1).

### Association between rs4410790 genotype and serum IgG levels

The rs4410790 TT genotype was significantly associated with elevated IgG levels compared with TC/CC genotypes in univariable analysis (p = 0.047) (Table 2). After adjusting for potential confounders, a significant association of TT genotype with a higher IgG level persisted (p = 0.04). After further adjusting for coffee consumption, the results did not change materially, with IgG levels higher for TT genotype compared to TC/CC genotype (p = 0.02).

**Table 2:** Adjusted mean levels of serum IgG by rs4410790 genotype.

		TT	TC/CC	Difference for TT vs. TC/CC	P value
IgG levels (arbitrary unit (A.U.))	Univariable	20278 (965)	17891 (701)	2387	0.047
	Multivariable model 1^1^	20362 (964)	17847 (695)	2515	0.04
	Multivariable model 2 ^2^	20626 (946)	17707 (680)	2919	0.02

*Data are presented as mean (standard error)

^1^Multivariable model 1 was adjusted for age, sex, alcohol consumption, coffee consumption, current overweight/obese, overweight/obese in high school, smoking status, aerobic exercise, 25(OH)D level.

^2^Multivariable model 2 was additionally adjusted for coffee consumption.

### Association between rs4410790 genotype and serum IgG levels by lifestyle factors

**Fig 1:**
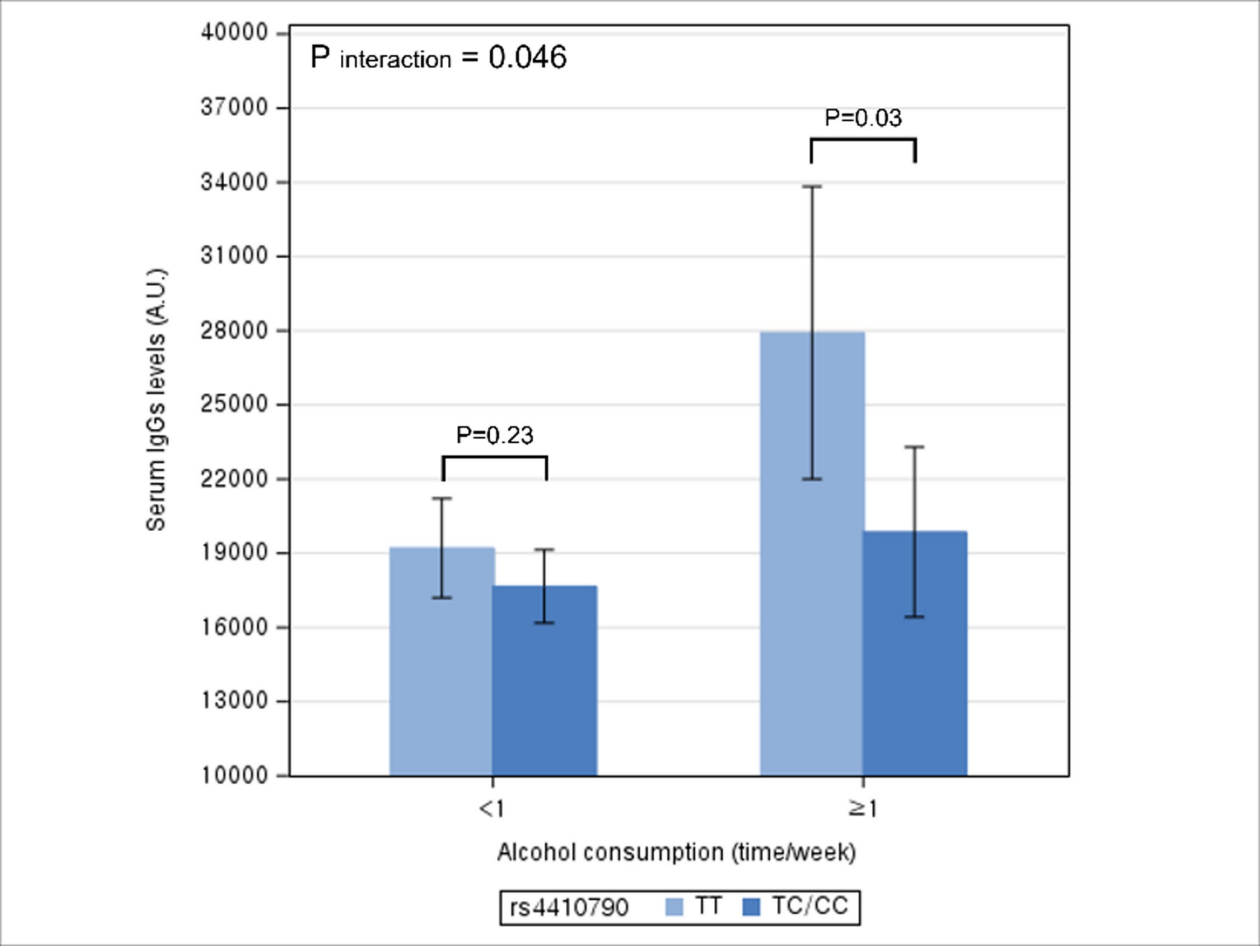
Multivariable association between rs4410790 genotype and serum IgG levels according to frequency of alcohol consumption. * The height of bar represents adjusted mean level of serum IgG and error bar represents its 95% confidence interval. * Difference in the adjusted mean levels of serum IgG levels between rs4410790 genotype represents the coefficient for the association between rs4410790 genotype and IgG levels.

By frequency of alcohol consumption, after adjusting for potential confounders, TT genotype had significantly higher serum IgG levels compared to TC/CC genotype among individuals drinking ≥ 1 time/week (p = 0.03), but not among individuals drinking<1 time/week (p = 0.23) (Fig 1). The interaction between rs4410790 genotype and frequency of alcohol consumption on serum IgG levels was statistically significant (P interaction = 0.046). Further adjustment of coffee consumption did not change the aforementioned results (S1 Table and S1 Fig).

**Fig 2:**
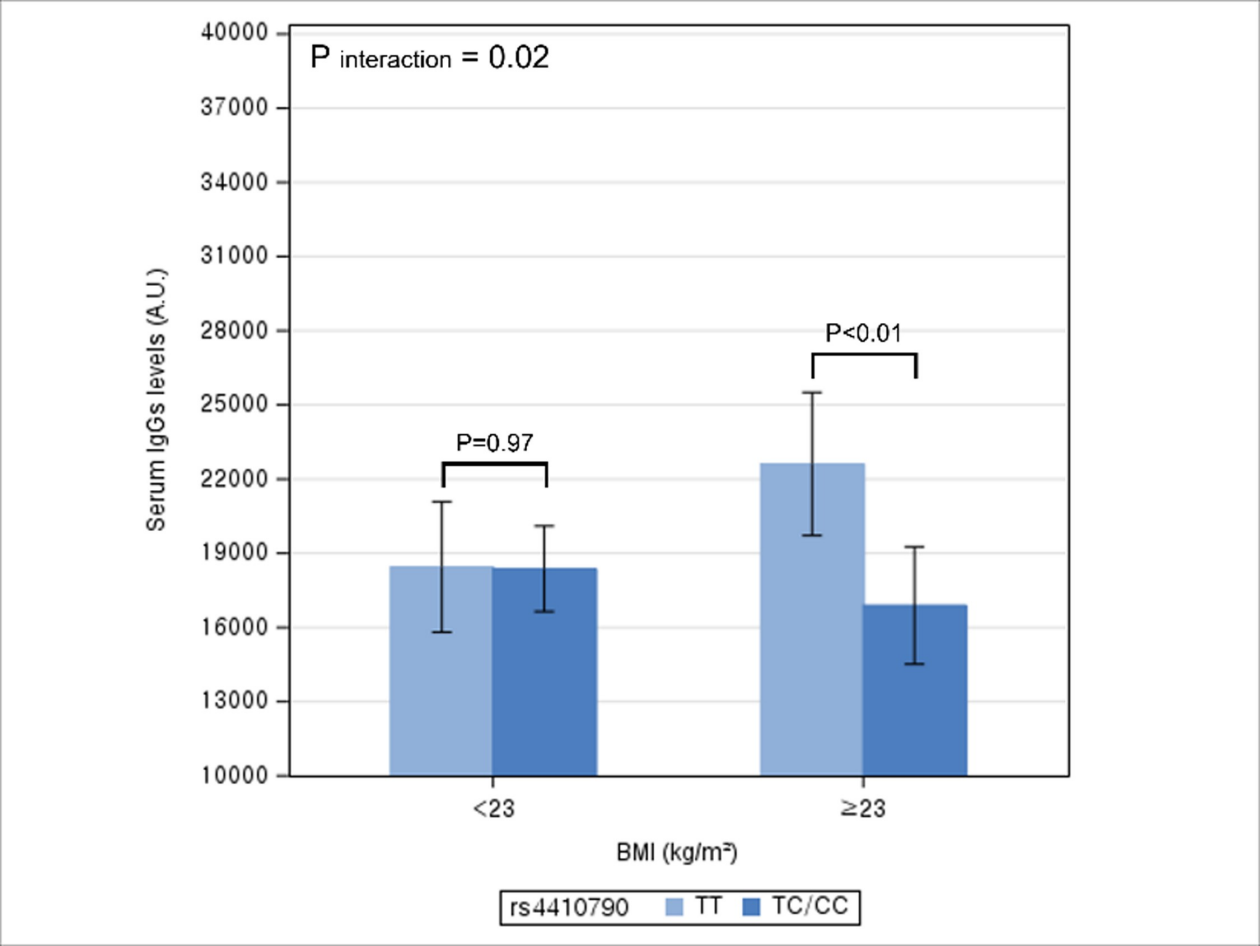
Multivariable association between rs4410790 genotype and serum IgG levels according to overweight/obese status. * The height of bar represents adjusted mean level of serum IgG and error bar represents its 95% confidence interval. * Difference in the adjusted mean levels of serum IgG levels between rs4410790 genotype represents the coefficient for the association between rs4410790 genotype and IgG levels.

By overweight/obese status, after adjusting for potential confounders, TT genotype had significantly higher serum IgG levels compared to TC/CC genotype among individuals with BMI≥23kg/m^2^ (p<0.01), but not among individuals with BMI <23kg/m^2^ (p = 0.97) (Fig 2). The interaction between rs4410790 genotype and overweight/obese on serum IgG levels was statistically significant (P interaction = 0.02). Additional adjustments for coffee intake did not lead to material changes in the previous results (S2 Table and S2 Fig).

**Fig 3:**
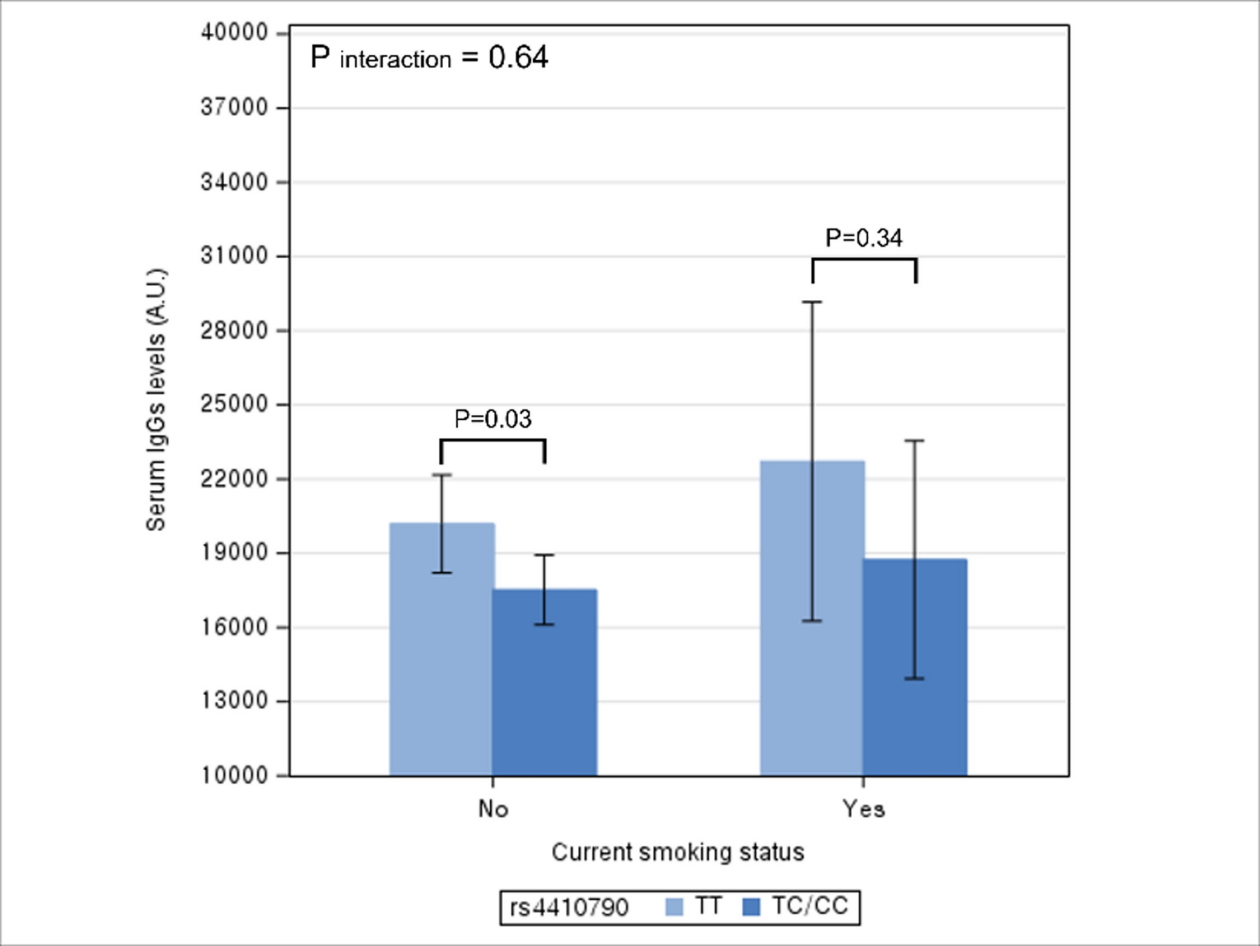
Multivariable association between rs4410790 genotype and serum IgG levels according to current smoking status. * The height of bar represents adjusted mean level of serum IgG and error bar represents its 95% confidence interval. * Difference in the adjusted mean levels of serum IgG levels between rs4410790 genotype represents the coefficient for the association between rs4410790 genotype and IgG levels.

By current smoking status, after adjusting for potential confounders, TT genotype had a significantly higher serum IgG levels compared to TC/CC genotype among no current smokers (p = 0.03), but not among current smokers (p = 0.34) (Fig 3). The interaction between rs4410790 genotype and smoking on the serum IgG level was not statistically significant (P interaction = 0.64). Additional adjustment of coffee consumption did not change the aforementioned results (S3 Table and S3 Fig).

**Fig 4:**
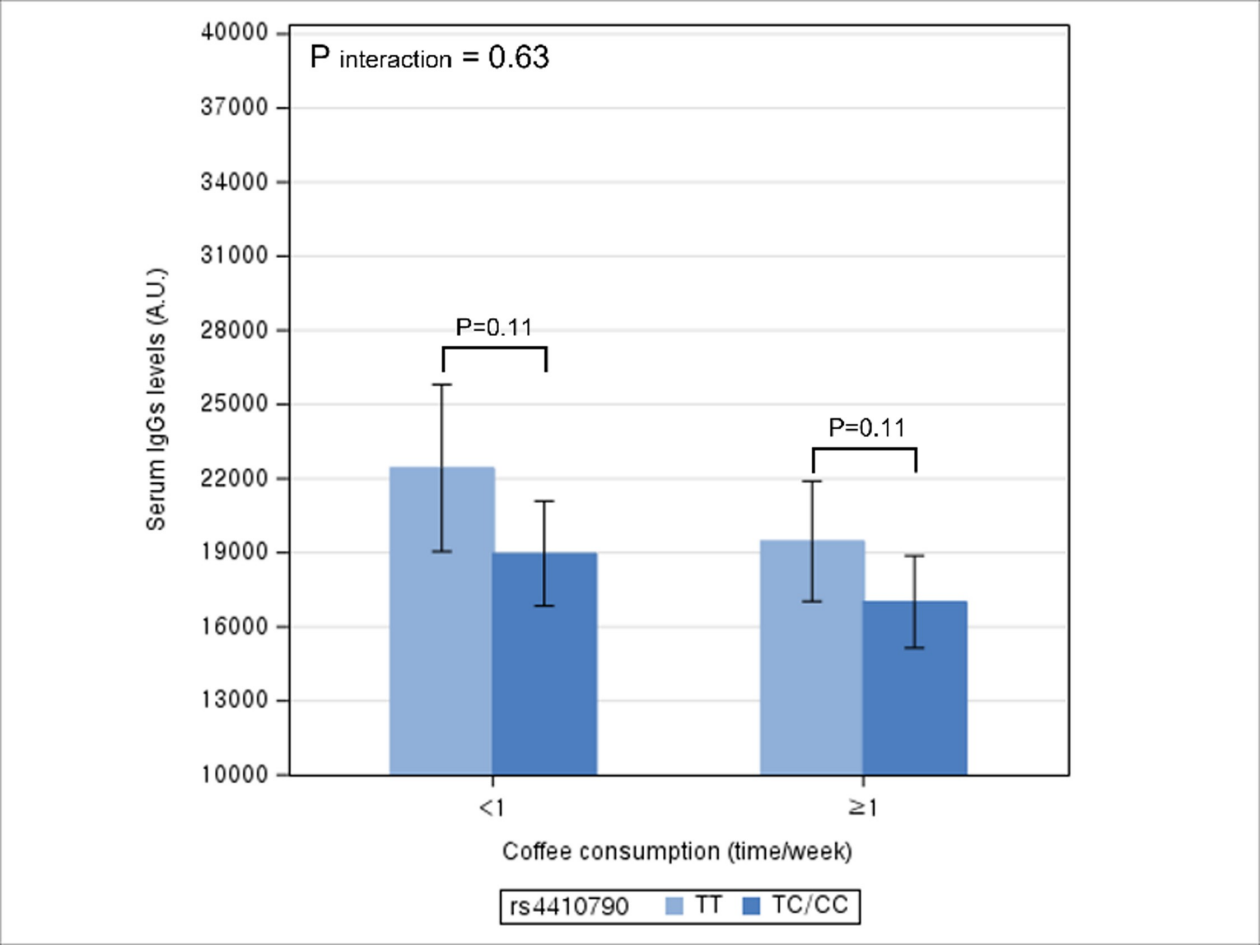
Multivariable association between rs4410790 genotype and serum IgG levels according to coffee consumption. * The height of bar represents adjusted mean level of serum IgG and error bar represents its 95% confidence interval.* Difference in the adjusted mean levels of serum IgG levels between rs4410790 genotype represents the coefficient for the association between rs4410790 genotype and IgG levels.

By frequency of coffee consumption, after adjusting for potential confounders, TT genotype had a not significantly higher serum IgG levels compared to TC/CC genotype among individuals coffee consumption≥1 time/week (p = 0.11), and also among individuals consumption <1 time/week (p = 0.11) (Fig 4). The interaction between rs4410790 genotype and coffee consumption on the serum IgG level was not statistically significant (P interaction = 0.63).

## Discussion

In this study, we observed an association between rs4410790 genotype and serum IgG levels, with the levels higher in the TT genotype than in TC/CC genotype. The relationship varied significantly by levels of alcohol consumption and overweight/obese status, but not by smoking status and coffee consumption. Specifically, higher IgG levels associated with the TT genotype were evident in frequent drinkers and individuals with BMI≥23kg/m,^2^ but not in their counterparts.

The variants of rs4410790 have been most widely investigated in relation to caffeine intake [5]. Caffeine is primarily metabolized by CYP1A2, which accounts for approximately 95% of caffeine clearance in the liver [6]. The AHR protein regulates CYP1A2, by inducing CYP1A2 transcription [6]. Thus, the *AHR* gene and protein are implicated in caffeine metabolism. In a genome-wide meta-analysis of 47,341 European descendants from five U.S. studies, rs4410790 has been identified to be the most strongly associated SNP for habitual caffeine consumption [5]. In a Costa Rican population of men and women with mean age of 57 years, non-carriers of C allele were associated with lower caffeine consumption (< 100 mg/day) compared to carriers of C allele (> 400 mg/day) [6]. Individuals who metabolize caffeine slowly are likely to consume less caffeine and thus, less coffee, a major dietary source of caffeine. Indeed, in a genome-wide meta-analysis of European and African American adults, non-carriers of C allele were associated with lower coffee consumption compared to carriers of C allele [5].

While no studies have investigated the relationship between coffee intake and IgG levels, coffee is known for its anti-inflammatory effect [18]. Considering that IgG level is a marker of chronic inflammation, individuals with rs4410790 TT genotype, via lower consumption of caffeine or coffee, are likely to have higher levels of inflammation and thus, higher IgG levels. However, the potential indirect effect of rs4410790 variants on IgG levels through caffeine/coffee intake is not supported by our findings. In our multivariable analyses to examine the relationship between rs4410790 genotype and IgG levels, additional adjustment of coffee consumption did not change the results, which provides evidence against the potential mediating role of caffeine/coffee consumption. Furthermore, the relationship between rs4410790 genotype and IgG levels did not vary significantly by the level of caffeine/coffee consumption. The observation that caffeine/coffee consumption is neither mediator nor modifier for the relationship suggests that a direct influence of *AHR* rs4410790 genotype on serum IgG levels may exist. Indeed, previous studies showed that *AHR* rs4410790 variant is associated with methylation of *AHR* gene [1], which is likely to be implicated in the regulation of immune system and inflammatory responses, as marked by IgG levels [12].

In our study, elevated IgG levels associated with the TT genotype were pronounced among frequent drinkers and individuals with BMI≥23kg/m^2^. When ethanol is metabolized, acetaldehyde and reactive oxygen species are produced, which activate inflammatory signaling pathways [26]. Chronic alcohol consumption also contributes to systematic inflammation by enhancing gut permeability to microflora-derived lipopolysaccharide, a cell all component of gram-negative bacteria that elicit inflammatory response [26]. Also, adipocytes are enriched in macrophages, which secret pro-inflammatory cytokines [16, 27]. Considering the established pro-inflammatory effects of alcohol and excessive fat [28–33], our results suggest that serum IgG levels may be influenced by synergistic effects of genetic and lifestyle factors. Individuals with TT genotype are genetically more prone to inflammation and such susceptibility may be readily manifest as an elevated IgG level when exposed to higher alcohol and adiposity, but be countered by avoiding alcohol intake and maintaining a healthy weight.

On the other hand, smoking status of individuals did not modify the relationship between rs4410790 genotype and serum IgG levels. While cigarette smoke contains multiple reactive oxygen species that stimulate the release of pro-inflammatory markers [15], the effects of smoking on immunity are complicated, inducing both pro-inflammatory and immune-suppressive effects [15]. Thus, relationships between smoking and multiple markers reflecting inflammatory processes do not always point to the same direction. For example, while C-reactive proteins, neutrophil extracellular trap, TNF-α and IL-6 increased significantly when individuals were exposed to smoking [34–36], serum IgG levels were higher among non-smokers compared with smokers [37]. Of note, 2,3,7,8-tetracholorodibenzo-p-dioxin, a major toxic component in cigarettes [38], functions as a ligand of AHR and is shown to suppress immunoglobin expression including IgG [12]. Yet, in our study consisting of young adults in early 20s, serum IgG levels were higher among smokers than non-smokers. The net effect of smoking on immunity depends on multiple factors (e.g., dose, type, and duration of smoking, host immunity) [15] and this complexity might have weakened potential interactions between smoking and genetic predisposition to higher IgG levels.

Our study has several strengths. First, in examining the relationship between rs4410790 variants and serum IgG levels, we controlled for the effects of age, sex, drinking, coffee consumption, smoking, overweight/obese, overweight/obese in high school, exercise, and 25(OH)D levels that have been suggested to influence serum IgG levels [17, 39–44]. Second, causal inference from a cross-sectional study is inherently limited due to reverse causality. Nonetheless, genetic variations are innate and thus, it is guaranteed in our study that rs4410790 variants precede serum IgG levels. Third, by providing evidence for the gene and environment interaction, our study suggests that genetic predisposition to higher IgG levels can be mitigated through lifestyle modifications such as drinking <1 time/week and maintaining healthy weight.

There are several limitations to acknowledge. First, as the first study that examined and identified an association between *AHR* rs4410790 variant and serum IgG levels, we cannot rule out a chance finding. Therefore, despite previous evidence for an association between rs4410790 variant and cerebellum *AHR* gene expression[1], the association between rs4410790 variant and serum IgG levels observed in our study could have been mediated by the effect of rs4410790 variant on nearby genes other than *AHR* gene that have influence on serum IgG concentrations. Third, considering that modest genetic predisposition tends to manifest at an early stage in life when major underlying diseases are unlikely, findings from our study population consisting of young adults may not be generalizable to older populations. Finally, our findings might be of less public health importance to other populations where the frequency of T allele for rs4410790 is relatively low (e.g., European, 38%) compared to 60% in our population [45].

## Conclusion

In summary, our study suggests that rs4410790 variant near the *AHR* gene was associated with serum IgG levels, a likely marker of chronic inflammation, in early adulthood. An elevation in serum IgG levels associated with rs4410790 TT genotype might be mitigated by healthy lifestyle factors such as infrequent drinking and healthy weight. As this is the first study that provided evidence for a relationship between rs4410790 genotype and serum IgG levels and for its effect modification by lifestyle factors that have been shown to be associated with IgG levels, further studies are warranted to validate our findings.

## Supporting information

S1 Table Multivariable association between rs4410790 genotype and serum IgG levels according to alcohol consumption. *Data are presented as means (standard error). ^1^Multivariable model 1 was adjusted for age, sex, alcohol consumption, coffee consumption, current overweight/obese, overweight/obese in high school, smoking status, aerobic exercise, 25(OH)D level. ^2^Multivariable model 2 was additionally adjusted for coffee consumption.
(DOCX)

S2 Table Multivariable association between rs4410790 genotype and serum IgG levels according to obesity status. *Data are presented as means (standard error) ^1^Multivariable model 1 was adjusted for age, sex, alcohol consumption, coffee consumption, current overweight/obese, overweight/obese in high school, smoking status, aerobic exercise, 25(OH)D level. ^2^Multivariable model 2 was additionally adjusted for coffee consumption.
(DOCX)

S3 Table Multivariable association between rs4410790 genotype and serum IgG levels according to current smoking status. *Data are presented as means (standard error) ^1^Multivariable model 1 was adjusted for age, sex, alcohol consumption, coffee consumption, current overweight/obese, overweight/obese in high school, smoking status, aerobic exercise, 25(OH)D level. ^2^Multivariable model 2 was additionally adjusted for coffee consumption.
(DOCX)

S4 Table Multivariable association between rs4410790 genotype and serum IgG levels according to coffee consumption. *Data are presented as means (standard error) ^1^Multivariable model 1 was adjusted for age, sex, alcohol consumption, coffee consumption, current overweight/obese, overweight/obese in high school, smoking status, aerobic exercise, 25(OH)D level.
(DOCX)

S1 Fig Multivariable association between rs4410790 genotype and serum IgG levels according to alcohol consumption, without adjusting for overweight/obese in high school. * The height of bar represents adjusted mean level of serum IgG and error bar represents its 95% confidence interval. * Difference in the adjusted mean levels of serum IgG levels between rs4410790 genotype represents the coefficient for the association between rs4410790 genotype and IgG levels.
(TIF)

S2 Fig Multivariable association between rs4410790 genotype and serum IgG levels according to overweight/obese status exclude adjusting overweight/obese in high school. * The height of bar represents adjusted mean level of serum IgG and error bar represents its 95% confidence interval. * Difference in the adjusted mean levels of serum IgG levels between rs4410790 genotype represents the coefficient for the association between rs4410790 genotype and IgG levels.
(TIF)

S3 Fig Multivariable association between rs4410790 genotype and serum IgG levels according to current smoking status exclude adjusting overweight/obese in high school. * The height of bar represents adjusted mean level of serum IgG and error bar represents its 95% confidence interval. * Difference in the adjusted mean levels of serum IgG levels between rs4410790 genotype represents the coefficient for the association between rs4410790 genotype and IgG levels.
(TIF)
