# Supplementary material for: AHR rs4410790 genotype and IgG levels: Effect modification by lifestyle factors
Source: PLoS One. 2023 Oct 2;18(10):e0290700. doi: 10.1371/journal.pone.0290700 (PMC10545101; doi:10.1371/journal.pone.0290700)
Supplement: S1 Table — *Data are presented as means (standard error). 1Multivariable model 1 was adjusted for age, sex, alcohol consumption, coffee consumption, current overweight/obese, overweight/obese in high school, smoking status, aerobic exercise, 25(OH)D level. 2Multivariable model 2 was additionally adjusted for coffee consumption. (DOCX) [file pone.0290700.s001.docx]

**S1 Table. Multivariable association between rs4410790 genotype and serum IgG levels according to alcohol consumption.**

|  | **Alcohol consumption** | **rs4410790** | **Adjusted mean**  **level of serum IgG*** | **Difference**  **for TT vs. TC/CC** | **P value** |
| --- | --- | --- | --- | --- | --- |
| Multivariable model 1^1^ | <1 | TT | 19208 | 1547 | 0.23 |
|  |  | TC/CC | 17661 |  |  |
|  | ≥1 | TT | 27918 | 8061 | 0.03 |
|  |  | TC/CC | 19857 |  |  |
| Multivariable model 2^2^ | <1 | TT | 19505 | 2008 | 0.11 |
|  |  | TC/CC | 17497 |  |  |
|  | ≥1 | TT | 28332 | 8630 | 0.03 |
|  |  | TC/CC | 19702 |  |  |

*Data are presented as means (standard error)

^1^Multivariable model 1 was adjusted for age, sex, alcohol consumption, coffee consumption, current overweight/obese, overweight/obese in high school, smoking status, aerobic exercise, 25(OH)D level.

^2^Multivariable model 2 was additionally adjusted for coffee consumption.
