# Supplementary figures and images for: AHR rs4410790 genotype and IgG levels: Effect modification by lifestyle factors
Source: PLoS One. 2023 Oct 2;18(10):e0290700. doi: 10.1371/journal.pone.0290700 (PMC10545101; doi:10.1371/journal.pone.0290700)

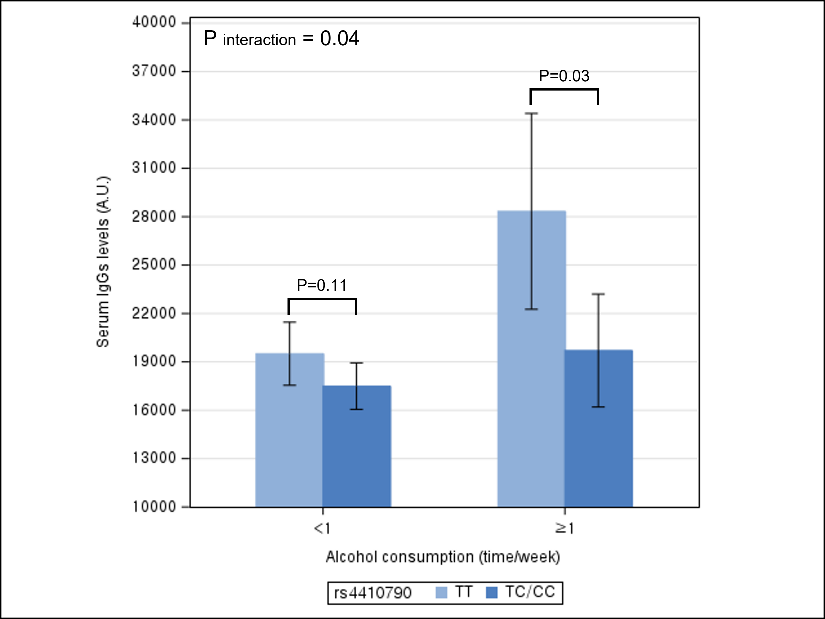

Supplement: S1 Fig — * The height of bar represents adjusted mean level of serum IgG and error bar represents its 95% confidence interval. * Difference in the adjusted mean levels of serum IgG levels between rs4410790 genotype represents the coefficient for the association between rs4410790 genotype and IgG levels. (TIF) [file pone.0290700.s005.tif]

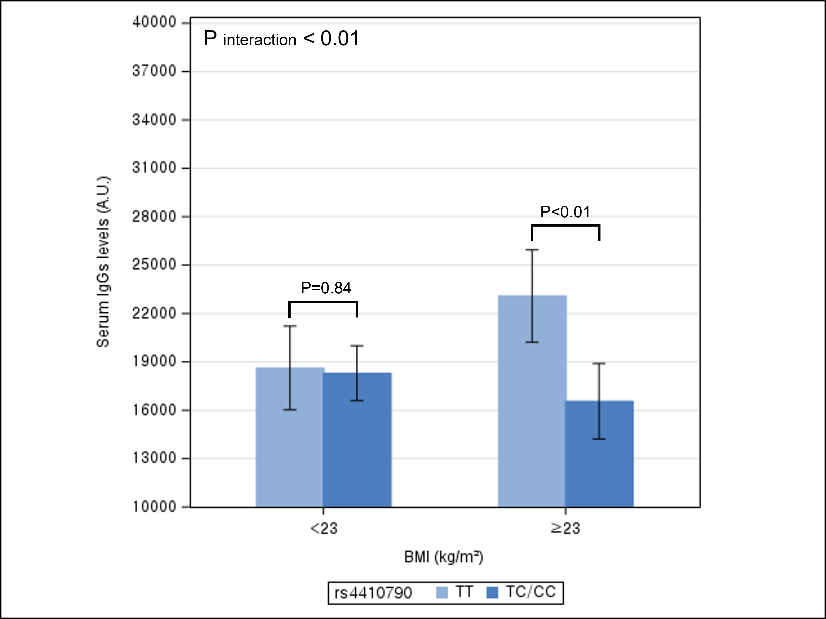

Supplement: S2 Fig — * The height of bar represents adjusted mean level of serum IgG and error bar represents its 95% confidence interval. * Difference in the adjusted mean levels of serum IgG levels between rs4410790 genotype represents the coefficient for the association between rs4410790 genotype and IgG levels. (TIF) [file pone.0290700.s006.tif]

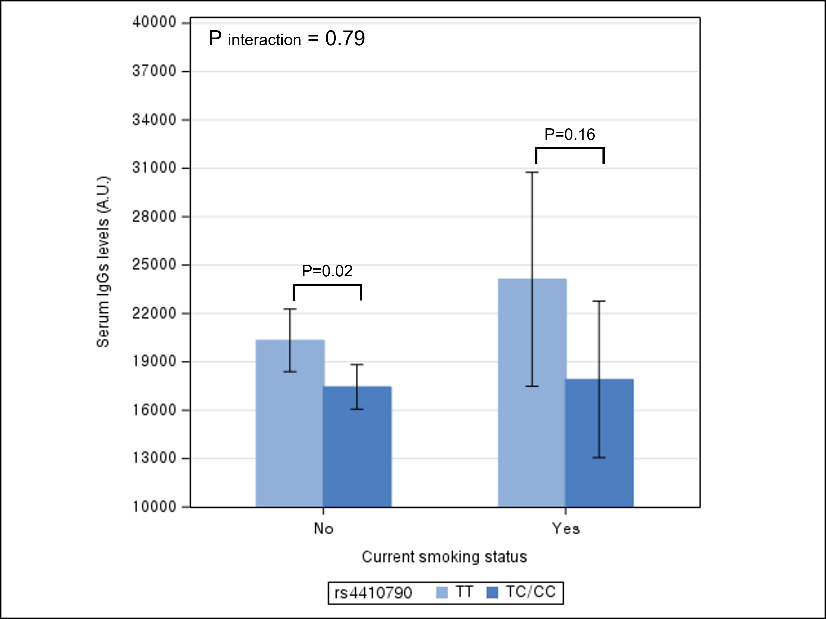

Supplement: S3 Fig — * The height of bar represents adjusted mean level of serum IgG and error bar represents its 95% confidence interval. * Difference in the adjusted mean levels of serum IgG levels between rs4410790 genotype represents the coefficient for the association between rs4410790 genotype and IgG levels. (TIF) [file pone.0290700.s007.tif]
